# Predictors of non-adherence to an early in-hospital rehabilitation program after surgery for hip fracture in a co-managed orthogeriatric unit

**DOI:** 10.1007/s40520-024-02857-w

**Published:** 2024-10-12

**Authors:** Roberto Presta, Enrico Brunetti, Valeria Quaranta, Silvio Raspo, Paola Cena, Giulia Carignano, Martina Bonetto, Chiara Busso, Gianluca Isaia, Marco Marabotto, Giuseppe Massazza, Mario Bo

**Affiliations:** 1 Department of Medical Sciences, University of Turin, Corso A. M. Dogliotti 14, Torino, 10126 Italy; 2 Division of Geriatrics, Department of General and Specialistic Medicine, Città della Salute e della Scienza University Hospital, Turin, Italy; 3 Department of Experimental and Clinical Medicine, University of Florence, Florence, Italy; 4 Division of Geriatrics, Department of Medical Specialties, Santa Croce e Carle Hospital, Cuneo, Italy; 5 Division of Physical Medicine and Rehabilitation, Department of Surgical Sciences, University of Turin, Turin, Italy

**Keywords:** Orthogeriatrics, Hip fracture, Early in-hospital rehabilitation, Non-adherence, Functional outcome, Comprehensive geriatric assessment

## Abstract

**Background:**

Hip fracture is a common event in older adults, leading to an increased risk of mortality, disability, and higher healthcare costs. Early in-hospital rehabilitation after surgery within orthogeriatric units may improve outcomes with limited incident complications even in the oldest old. We aimed to determine the prevalence and predictors of non-adherence to early rehabilitation in the orthogeriatric unit of an Italian tertiary hospital and its impact on outcomes and setting at discharge.

**Methods:**

Retrospective observational single-centered cohort study. Patients aged ≥ 65 years admitted to the orthogeriatric unit for hip fracture who underwent surgery between April 2019 and October 2020 were considered eligible if able to walk autonomously or with assistance and independent in at least 2 Basic Activities of Daily Living. Along with sociodemographic and geriatric variables, characteristics of surgery and rehabilitation, in-hospital complications and functional outcomes at discharge were collected. The primary outcome was non-adherence to the early in-hospital rehabilitation program.

**Results:**

Among 283 older patients (mean age 82.7 years, 28.6% male), non-compliance with physical therapy was assessed in 49 cases (17.3%), characterized by worse pre-fracture clinical, cognitive, and functional status and showing worse outcomes in terms of mobilization at discharge. After multivariable analysis, non-adherence was independently associated with the onset of delirium (OR 5.26, 95%CI 2.46–11.26; *p* < 0.001) or infections after surgery (OR 3.26, 95%CI 1.54–6.89; *p* < 0.001) and a systolic blood pressure at admission < 120 mmHg (OR 4.52, 95%CI 1.96–10.43, *p* < 0.001).

**Conclusions:**

Pre-fracture poor cognitive and functional status, along with lower systolic blood pressure, seem to make some patients more vulnerable to in-hospital complications (mainly delirium and infections) and negatively affect the adherence to physical therapy and, by consequence, clinical outcomes of rehabilitation.

## Introduction

Hip fracture is a common traumatic event in older adults, whose incidence increases progressively with age [1]. In the coming years, the absolute number of hip fracture in this segment of the population is expected to steadily rise as well as the average age of patients admitted to geriatric acute care units and rehabilitation centers, requiring an overall dissemination and improvement of standard geriatric and multidisciplinary competences across different clinical settings [2].

These fractures can lead to an increased risk of mortality, disability, clinical complications, and high healthcare costs [1, 3]. Despite their greater risk of poor clinical and functional outcomes, there is emerging evidence that a careful and multidisciplinary approach may improve the achievement of satisfying clinical and functional outcomes with limited incident complications even in the oldest old [4, 5]. Although models of multidisciplinary co-management are highly heterogeneous with different key elements and outcome parameters [2, 6], orthogeriatric units – usually involving geriatricians, physiatrists, orthopedic surgeons, and anesthesiologists, as well as physical therapists, nurses, and nutritionists [7, 8] – should aim to (i) restore the pre-fracture status as soon as possible, (ii) reduce complications, readmissions, and mortality rates, (iii) provide best value of care to the health system and (iv) initiate secondary fracture prevention [9, 10]. The amount of evidence reporting rehabilitation outcomes among older patients is increasing and national intersociety guidelines have been published in recent years, but there is currently no final consensus about the best possible treatment model [11].

In general, early mobilization and physical rehabilitation of patients with hip fracture, starting the same day or the day after surgery, is a crucial part of interventions in this setting, promoting shorter hospital stay [12, 13], lower rates of clinical complication [14, 15], better post-discharge physical function [16] and, ultimately, lower mortality [17]. However, the rehabilitation plan should be carefully tailored to each patient upon the assessment by the multidisciplinary team [11], accounting for clinical and cognitive status, timing, and type of surgery, which have emerged as key variables to maximize outcomes [18–20].

However, results of this intervention are quite heterogeneous in real-world patients, and such differences might be accounted for by several patients’ related factors, including pre-fracture level of functional and cognitive impairment, in-hospital complications, and attitude to the rehabilitation program. Most studies in this field focus on in-hospital rehabilitation outcomes after hip fracture [20–22], and to our knowledge, only a few studies have specifically explored the adherence of older patients to rehabilitation programs in co-managed orthogeriatric units, with non-adherence observed in approximately one out of four patients [23].

We hypothesized that non-compliance with in-hospital rehabilitation might be determined both by pre-fracture status and clinical peri- and post-operative complications and may represent a proxy variable of poor mobilization outcomes. Thus, the present study aimed to determine the prevalence and predictors of non-adherence to early in-hospital rehabilitation in patients admitted to an Italian orthogeriatric unit and its impact on outcomes and setting at discharge.

## Materials and methods

### Study design and setting

This retrospective observational single-centered cohort study was carried out at the Santa Croce e Carle hospital in Cuneo, northern Italy, where the orthogeriatric program was established since 2002 for all patients aged 65 years or older admitted to the orthopedic unit for any fracture. The orthogeriatric multidisciplinary team include orthopedics and geriatricians, as well as anesthetists, physiatrists and physical therapists, and the geriatrician acts as a consultant both before and after surgery.

The study was conducted according to the Recommendations Guiding Physicians in Biomedical Research Involving Human Subjects, approved by the local Ethics Committee and reported conforming to the Strengthening The Reporting of Observational Studies in Epidemiology Statement [24].

### Data collection

Patients aged 65 years or older hospitalized in the orthopedic unit between April 2019 and October 2020 (with a suspension from March to June 2020 due to SARS-CoV-2 pandemics) who underwent surgery after hip fracture were considered eligible if able to walk autonomously or with assistance and independent in at least 2 functions upon Katz’s Basic Activities of Daily Living scale (BADL) [25] before fracture. Signed informed consent was obtained from all patients. Data were retrospectively collected from medical records using standardized evaluation protocols by a resident doctor from the local section of Geriatrics under the supervision of senior specialists in Geriatrics and Physiatrics.

At the time of admission, age, sex, marital status and living situation (i.e., whether the patient lives with his/her spouse, alone, with other family members, with home caregiver or in a long-term care facility) were recorded.

Characteristics of hip fracture (i.e., intracapsular, pertrochanteric, diaphyseal or periprosthetic), hospitalization (i.e., elective or urgency), surgical technique (i.e., osteosyntheses, hemi-arthroplasty or total hip arthroplasty) and anesthesia (i.e., general or spinal), as well as the time from fracture to surgery were collected.

The peri-operative need for blood transfusion and the presence of post-operative hypotension and complications (i.e., in-hospital infection, delirium, acute urinary retention, pressure ulcers, acute respiratory and heart failure) were registered.

Among rehabilitation outcomes, both verticalization at any time of in-hospital rehabilitation and mobilization status at discharge (i.e., ability to walk with assistance, verticalization, in wheelchair or bedridden) were noted. Length of stay and discharge destination (e.g., at home with or without nurse assistance, nursing homes, rehabilitation/long-term care facilities, others) were also recorded.

### Standardized multidimensional assessment

Each patient underwent a routine multidimensional geriatric evaluation at admission. Functional status was assessed using Katz’s BADL (range 0–6, with scores ≥ 2 identifying functional dependence) [25] and a modified version of the Instrumental Activities of Daily Living scale (IADL, range 0–14, with scores < 10 identifying partial or absence of autonomy) [26]. Cognitive status was evaluated using the Short Portable Mental Status Questionnaire (SPMSQ, range 0–10, moderate to severe cognitive impairment if ≥ 5) [27]. Disease burden and severity were evaluated using the Cumulative Illness Rating Scale (CIRS) [28]. This tool rates the severity of diseases (range 1–5, with 1 indicating no affection and 5 severe impairment) according to major organ groups, hypertension, and psychiatric/behavioral area for a total of 14 items. From CIRS ratings, the composite measures of Comorbidity Index (CIRS-CI) and Severity Index (CIRS-SI) are derived, representing respectively the number of items scoring 3 or more and the mean of CIRS items, both excluding the psychiatric/behavioral category [28]. The presence of hypertension and dementia at admission was also recorded.

### Intervention and outcome variables

According to national recommendations [11], early in-hospital rehabilitation program consisted of daily individualized progressive high-intensity structured 30-minutes training on weekdays, including weight bearing, strength, gait, and balance exercises. In our orthogeriatric unit, the rehabilitation plan is drawn up by the physiatrist in charge on the day of surgery. Accordingly, the following day the patient’s rehabilitation program starts, and the physical therapist notes the progress or the lack of participation for any cause (i.e., complications, refusal, pain, etc.) in the medical chart at the end of each daily training. Non-adherence to the early in-hospital rehabilitation program represented the primary outcome of the study and was retrospectively evaluated if non-compliance with physical exercise was noted by the therapist for at least 20% of rehabilitation sessions throughout the hospital stay, based on previous experience [29].

### Statistical analysis

For the expected prevalence non-adherence to in-hospital rehabilitation after hip fracture surgery of 24%^23^, the required sample size was 282 for the margin of error or absolute precision of 5% in estimating the prevalence with 95% confidence. This sample size was calculated using the ScalaR SP [30].

Statistical analysis was performed using SPSS software (IBM SPSS Statistics, version 28.0.1.0). The absolute and relative frequencies of dichotomous and categorical variables were calculated, as well as the mean and standard deviation or median and 25° and 75° percentiles for normally distributed and not normally distributed continuous variables, as appropriate. Prevalence of non-adherence and its exact 95% confidence interval (CI) were calculated. Univariate analysis of potential association between baseline variables and adherence to physical therapy was conducted using the analysis of variance for normally distributed continuous variables, the Mann-Whitney test for not normally distributed continuous variables and the Chi square test for dichotomous and categorical variables. Clinically relevant variables were then introduced in a multivariable logistic regression model, with non-adherence to physical therapy as the dependent variable, and adjusted odds ratios (OR) and their 95% CIs were calculated. Significance level was set at α < 0.05 for all tests.

## Results

During the study period, 606 patients aged 65 years or older were hospitalized for any type of fracture, of which 345 for hip fracture; 62 were not enrolled because they were unable to walk and dependent in more than 4 functions upon Katz’s scale before fracture. A final sample of 283 patients was thus included in the analysis.

The main characteristics of the overall study sample (mean age 82.7 [7.9] years, 28.6% male) are presented in Table 1. Most of them lived at their home (258, 91.2%), around one-half was dependent in at least one BADL (132, 46.6%), partially or not autonomous in IADL (138, 48.8%) and was prescribed with 5 or more drugs (138, 48.8). More than one-third used to ambulate with assistance (108, 38.7%) and one out of four had known diagnosis of dementia (72, 25,4%), with 40 patients (14.1%) suffering from moderate to severe cognitive impairment. At admission, hypertension was present and treated in 198 patients (70.0%), whereas one out of five had systolic blood pressure below 120 mmHg (56, 19.8%), with an average systolic and diastolic pressure of 140.6 (21.7) and 72.5 (12.6) mmHg, respectively; no baseline variable turned out to differ between patients with and without systolic hypotension at admission.

**Table 1:** Characteristics of the of the overall sample and by outcome

	Overall sample (*n* = 283)		Adherence to rehabilitation (*n* = 234)		Non-adherence to rehabilitation (*n* = 49)		*p*
**Patients’ characteristics**							
Age (years), mean (SD)	82.7	(7.9)	82.3	(7.9)	84.4	(7.6)	ns
Sex male, *n* (%)	81	(28.6)	65	(27.8)	16	(32.7)	ns
Marital status							
Widow(er), *n* (%)	140	(49.5)	115	(49.1)	25	(51.0)	ns
Married, *n* (%)	106	(37.5)	88	(37.6)	18	(36.7)	ns
Single, *n* (%)	30	(10.6)	24	(10.3)	6	(12.2)	ns
Divorced, *n* (%)	7	(2.5)	7	(3.0)	0	(0)	ns
Living situation							
At home, with assistance, *n* (%)	144	(50.9)	109	(47.2)	35	(72.9)	< 0.001
At home, independent, *n* (%)	114	(40.3)	107	(46.3)	7	(14.6)	< 0.001
At a nursing home, *n* (%)	21	(7.4)	15	(6.5)	6	(12.5)	ns
**Functional autonomy and comorbidities at admission**							
BADL lost functions ≥ 1 (dependent), *n* (%)	132	(46.6)	99	(42.3)	33	(67.3)	< 0.001
IADL ≤ 9 (partially or not autonomous), *n* (%)	162	(57.2)	121	(51.7)	41	(83.7)	< 0.001
CIRS – Comorbidity index, median (25°-75°)	3	(2–4)	3	(2–4)	3	(2–4)	ns
CIRS – Severity index, median (25°-75°)	1.5	(1.4–1.7)	1.5	(1.3–1.7)	1.5	(1.4–1.7)	ns
≥ 5 drugs at admission, *n* (%)	138	(48.8)	115	(49.1)	23	(46.9)	ns
SPMSQ ≥ 5 errors (moderate-severe cognitive impairment), *n* (%)	40	(14.1)	20	(8.5)	20	(40.8)	< 0.001
Dementia, *n* (%)	72	(25.4)	45	(19.2)	27	(55.1)	< 0.001
Ambulation with assistance, *n* (%)	108	(38.7)	81	(34.6)	27	(55.1)	< 0.001
Hypertension, *n* (%)	198	(70.0)	168	(71.8)	30	(61.2)	ns
Systolic BP (mmHg), mean (DS)	140,6	(21,7)	141.9	(21.5)	134.0	(21.7)	0.020
Systolic BP < 120 mmHg, *n* (%)	56	(19.8)	39	(16.7)	17	(34.7)	0.007
Diastolic BP (mmHg), mean (DS)	72,5	(12,6)	72.7	(12.8)	71.3	(11.4)	ns
Hemoglobin (g/dL), median (25°-75°)	12,7	(11,7–13,5)	12,6	(11,4–13,4)	12,2	(11,5–13,1)	ns
sCr (mg/dL), median (25°-75°)	0,8	(0,7 − 1)	0,8	(0,7 − 1,1)	0,8	(0,6 − 1,1)	0.041
Ferritin (ng/mL), median (25°-75°)	188,6	(108,3-266,2)	181,2	(89,1-250,5)	213,4	(141,8-454,8)	0.001
**Characteristics of surgery**							
Type of fracture							
Pertrochanteric, *n* (%)	161	(56.9)	129	(55.1)	32	(65.3)	ns
Intracapsular, *n* (%)	83	(29.3)	74	(31.6)	9	(18.4)	ns
Periprosthetic, *n* (%)	26	(9.2)	21	(9.0)	5	(10.2)	ns
Diaphyseal, *n* (%)	13	(4.6)	10	(4.3)	3	(6.1)	ns
Time fracture-surgery < 48 h, *n* (%)	203	(71.7)	167	(71.4)	36	(73.5)	ns
Type of surgery							
Osteosyntheses, *n* (%)							
Parallel implants, *n* (%)	10	(3.5)	8	(3.4)	2	(4.1)	ns
Sliding hip screw, *n* (%)	18	(6.4)	14	(6.0)	4	(8.2)	ns
Intramedullary nail, *n* (%)	187	(66.1)	151	(64.5)	36	(73.5)	ns
Hemi-arthroplasty, *n* (%)	35	(12.4)	32	(13.7)	3	(6.1)	ns
Total hip arthroplasty, *n* (%)	33	(11.7)	29	(12.4)	4	(8.2)	ns
Spinal anesthesia, *n* (%)	229	(80.9)	191	(81.6)	38	(77.6)	ns
Bleeding requiring blood transfusion, *n* (%)	139	(49.1)	108	(46.2)	31	(63.3)	0.043
**Characteristics of hospitalization**							
Time surgery-rehabilitation, median (25°-75°)	1	(1–2)	1	(1–2)	1	(1–3)	ns
Days of rehabilitation suspension, median (25°-75°)	2	(2–3)	2	(2–3)	2	(2–4)	0.001
Patients suffering ≥ 1 complication, *n* (%)	171	(60.4)	128	(54.7)	43	(87.8)	< 0.001
Infections, *n* (%)	87	(30.7)	63	(26.9)	24	(49.0)	0.004
UTIs, *n* (%)	42	(14.8)	35	(15.0)	7	(14.3)	ns
Pneumonia, *n* (%)	28	(9.9)	17	(7.3)	11	(22.4)	0.003
Delirium, *n* (%)	84	(29.7)	52	(22.2)	32	(65.3)	< 0.001
Acute respiratory failure, *n* (%)	40	(14.1)	32	(13.7)	8	(16.3)	ns
Acute heart failure, *n* (%)	23	(8.1)	17	(7.3)	6	(12.2)	ns
Pressure ulcers, *n* (%)	19	(6.7)	12	(5.1)	7	(14.3)	0.044
Acute urinary retention, *n* (%)	14	(4.9)	8	(3.4)	6	(12.2)	0.026
Post-surgery hypotension (≤ 90/60 mmHg), *n* (%)	95	(33.6)	76	(32,5)	19	(38,8)	ns
**Outcome at discharge**							
Systolic BP (mmHg), mean (SD)	128,3	(21,2)	129.4	(21.1)	123.1	(21.2)	ns
Diastolic BP (mmHg), mean (SD)	66	(12,1))	66.6	(11.5)	63.1	(14.6)	ns
Not verticalized patients, *n* (%)	28	(9.9)	13	(5.6)	15	(30.6)	< 0.001
Mobilization at discharge							
Able to walk with crutches, *n* (%)	13	(4.6)	13	(5,6)	0		ns
Able to walk with the physical therapist, *n* (%)	158	(55.8)	146	(62.4)	12	(24.5)	< 0.001
Verticalized, *n* (%)	82	(29.0)	61	(26.1)	21	(42.9)	< 0,001
In wheelchair, *n* (%)	25	(8.8)	13	(50.6)	12	(24.5)	< 0.001
Bedridden, *n* (%)	5	(1.8)	1	(0.4)	4	(8.2)	< 0.001
Length of stay (days), median (25°-75°)	9	(8–13)	10	(7.5–14)	9	(8–13)	ns
Setting of discharge							
Rehabilitation facilities, *n* (%)	130	(45.9)	114	(48.7)	16	(32.7)	< 0,001
at lower intensity, *n*	105	(80,7)	91	(38.9)	14	(28.6)	ns
at higher intensity, *n*	25	(19,3)	23	(9.8)	2	(4.1)	ns
Long-term care, *n* (%)	95	(33.6)	72	(30 − 8)	23	(46.9)	< 0,001
Home, *n* (%)	46	(16.3)	40	(17.1)	6	(12.2)	ns
Nursing home, *n* (%)	10	(3.5)	7	(3.0)	3	(6.1)	ns

BADL: Basic Activities of Daily Living; BP: blood pressure; CIRS: Cumulative Illness Rating Scale; IADL: Instrumental Activities of Daily Living; sCr: serum creatinine; SD: standard deviation; SPMSQ: Short Portable Mental State Questionnaire; UTI: urinary tract infection

Pertrochanteric fracture was the most common type of hip fracture (161, 56.9%) and osteosynthesis with intramedullary nail was the most frequent type of surgery (187, 66.1%), performed within 48 h from the fracture in 203 patients (71.7%). Almost one-half suffered from bleeding requiring blood transfusion during surgery (139, 49.1%), whereas 171 patients suffered from at least one medical complication (60.4%), mainly infections (87, 30.7%) and delirium (84, 29.7%), and one-third had post-surgery hypotension (95, 33.6%).

Rehabilitation generally started the day after surgery, with a median time of suspension for any reason of 2 days (2–3). During hospitalization, verticalization was not achieved in 28 patients (9.9%). At discharge, almost four out of five patients were transferred to rehabilitation or long-term facilities (225, 79.5%): most patients were able to walk with the assistance of the physical therapist (158, 55.8%) or with orthopedic devices (13, 4.6%), whereas 82 patients were only able to stand (29.0%), and 30 patients were on wheelchair or bedridden (10.6%).

Non-compliance with the early in-hospital rehabilitation program was assessed in 49 patients (17.3%, 95%CI 13.1–22.2%). In Table 1 the distribution of variables according to compliance to physical therapy is also reported. At univariate analysis, non-adherent patients were more dependent in BADL (67.3% vs. 42.3%, *p* < 0.001) and less autonomous in IADL (83.7% vs. 51.7%, *p* < 0.001), with more cognitive impairment (55.1% vs. 19.2%, *p* < 0.001), more frequently needing assistance in walking (55.1% vs. 34.6%, *p* < 0.001) and with lower systolic blood pressure at admission. No difference in the type of fracture or surgery or in the timing from fracture to surgery and from surgery to rehabilitation was observed between the two groups, whereas non-compliant patients suffered more frequently from bleeding requiring transfusion (63.3% vs. 46.2%, *p* = 0.043) and from at least one medical complication (87.8% vs. 54.7%, *p* < 0.001), mainly pneumonia, delirium, pressure ulcers and acute urinary retention. Mobilization at discharge was lower among non-compliant patients and around three out of four of them were unable to walk.

After multivariable analysis, non-adherence to the early in-hospital rehabilitation program was positively associated with a systolic blood pressure < 120 mmHg at admission (OR 4.52, 95%CI 1.96–10.43, *p* < 0.001) and the onset of delirium (OR 5.26, 95%CI 2.46–11.26; *p* < 0.001) and infection after surgery (OR 3.26, 95%CI 1.54–6.89; *p* < 0.001) (Table 2).

**Table 2:** Variables associated with non-adherence to early in-hospital rehabilitation programs: results of the multivariate logistic regression model*

Variables	Odds ratio (95% CI)		*p*
Systolic BP at admission < 120 mmHg	4.52	(1.96–10.43)	< 0,001
Delirium	5.26	(2.46–11.26)	< 0,001
Infections after surgery	3.26	(1.54–6.89)	< 0,001

* The following variables were included in the present model: age, sex, IADL (preserved functions), SPMSQ (number of errors), ≥ 1 medical complication, infection, delirium, systolic BP at admission < 120 mmHg, sCr at admission

BP: blood pressure; IADL: Instrumental Activities of Daily Living; sCr: serum creatinine; SPMSQ: Short Portable Mental State Questionnaire

## Discussion

This retrospective observational study aimed to assess the prevalence and predictors of non-adherence to an early in-hospital rehabilitation program among older patients undergoing hip fracture surgery within an Italian orthogeriatric unit. In our single-center sample of patients mostly living at home and able to walk autonomously or with assistance, with moderate functional autonomy, our findings showed that (i) in 71.5% of cases surgery was performed within 48 h from the fracture, as recommended by international guidelines, with spinal anesthesia most used (80.9%), and the rehabilitation program generally started the day after surgery; (ii) 17.3% of patients were deemed non-compliant with physical therapy, and these patients had worse pre-fracture clinical, cognitive and functional status and worse outcomes in terms of mobilization at discharge; (iii) after multivariable analysis, besides the onset of delirium or infections during hospitalization, a systolic blood pressure at admission < 120 mmHg was independently associated with non-adherence to our early in-hospital rehabilitation program. In summary, our results seem to suggest that pre-fracture poor cognitive and functional status make some patients more vulnerable to in-hospital complications (delirium and infections) which negatively affect the clinical outcomes of rehabilitation.

Previous studies and clinical guidelines on hip fracture management have already highlighted the main determinants of functional outcomes after early rehabilitation (e.g., age, sex, time between hospital admission and surgery, degree of surgical risk, pre-fracture functional and cognitive status and social support, pre-surgery complications, rehabilitation plan) [31, 32]. For these reasons, a multidisciplinary team of geriatricians, orthopedics, physiatrists, physical therapists, and specialized nurses are needed to properly manage these patients and achieve better outcomes [11].

However, most evidence explored variables associated with functional outcomes after hip fracture but, to our knowledge, our research is one of the first studies investigating the predictors of non-adherence to early in-hospital rehabilitation programs. Hulsbæk et al. mentioned that older age, a greater burden of comorbidities and lower pre-fracture function and cognitive status were associated with failing to complete physical therapy on the first postoperative day [20]. More specifically, Rosas Hernández et al. observed a relatively high adherence to a standardized rehabilitation program among old patients with acute hip fracture in a Spanish University hospital (76.0%), independently associated with living at home, functional autonomy, absence of cognitive impairment and a lower burden of comorbidities [23]. Similarly, in our sample, 82.7% were adherent to the early rehabilitation program. Although non-compliant patients were characterized by worse pre-fracture functional, physical, and cognitive status, after multivariable analysis non-adherence was independently associated with a systolic blood pressure < 120 mmHg at admission and more frequent medical complications such as delirium and infections after surgery. As expected, non-adherent patients presented worse functional outcomes and were more frequently institutionalized. This evidence, in addition to offering physical therapists a valuable reference for estimating each patient’s adherence to the rehabilitation program, reinforces the importance of careful clinical management of elderly patients during their hospital stay for hip fracture, particularly within the orthogeriatric unit. It suggests the need for appropriate modifications of risk factors to better prevent medical complications such as hypotension, delirium, and infections, which significantly impact functional outcomes in these patients [33–37].

Some limitations of the present study should be addressed. Although consistent with similar works, our single-center study reflects the reality of a definite geographical area and of a specific orthogeriatric model. In consideration of the great heterogeneity of care pathways for older patients with hip fracture among different countries and national areas, a multicenter design is currently difficult to perform. For this reason, along with the individualized training program adopted in our Center, the generalizability of our findings is inevitably limited. Indeed, the clinical and socio-economic implications of dedicated multidisciplinary care models for these patients should suggest the impellent need for a wide implementation and standardization at national level.

Furthermore, the merely numerical criteria we used to identify non-adherence, although already adopted elsewhere, might not reflect the global impact of in-hospital rehabilitation sessions in these patients [29]. A few observational methods have been devised to measure patients’ adherence behaviors during in-hospital physiotherapy, including subjective physician’s assessment through behavioral observations resulting in an average adherence percentage [38] but, to our best knowledge, there is no evidence of the reliability of these subjective adherence measures.

At the same time, our sample could be considered a population-based sample, since it likely reflects all patients with hip fracture in a large area of our region where our Center is the only major public hospital, and its size, although relatively limited, is comparable to similar previous studies.

## Conclusions

In our sample of older patients undergoing hip fracture surgery within an Italian orthogeriatric unit, non-adherence to physical therapy, although present in a limited portion of our sample, represent a main determinant of worse rehabilitation outcomes and is mainly correlated with pre-fracture geriatric characteristics and lower blood pressure as well as common in-hospital complications such as delirium and infections. A multidisciplinary clinical management approach, based on a comprehensive geriatric assessment, may help to better prevent common clinical complications and optimize functional outcomes in these patients.

## Data Availability

All Authors had all access to the data in this work and approved the submission of the present manuscript. All material in this assignment is Authors’ own work and does not involve plagiarism. The data that support the findings of this study are available from the corresponding Author upon reasonable request.
